# Spatiotemporal dynamics and recurrence of chikungunya virus in Brazil: an epidemiological study

**DOI:** 10.1016/S2666-5247(23)00033-2

**Published:** 2023-04-06

**Authors:** William M de Souza, Shirlene T S de Lima, Leda M Simões Mello, Darlan S Candido, Lewis Buss, Charles Whittaker, Ingra M Claro, Nilani Chandradeva, Fabiana Granja, Ronaldo de Jesus, Poliana S Lemos, Daniel A Toledo-Teixeira, Priscilla P Barbosa, Antonio Carlos L Firmino, Mariene R Amorim, Larissa M F Duarte, Ivan B Pessoa, Julia Forato, Irihane L Vasconcelos, Ana Carolina B M Maximo, Emerson L L Araújo, Liana Perdigão Mello, Ester C Sabino, José Luiz Proença-Módena, Nuno R Faria, Scott C Weaver

**Affiliations:** Department of Microbiology and Immunology (W M de Souza PhD, Prof S C Weaver PhD), World Reference Center for Emerging Viruses and Arboviruses (W M de Souza, Prof S C Weaver), and Institute for Human Infections and Immunity (Prof S C Weaver), University of Texas Medical Branch, Galveston, TX, USA; Laboratório Central de Saúde Pública do Ceará, Fortaleza, Brazil (S T S de Lima PhD, L M Simões Mello MSc, A C L Firmino BSc, L M F Duarte BSc, I B Pessoa Jr BSc, I L Vasconcelos BSc, A C B M Maximo MSc, L Perdigão Mello BSc); Laboratory of Emerging Viruses, Department of Genetics, Microbiology and Immunology, Institute of Biology (S T S de Lima, F Granja PhD, D A Toledo-Teixeira MSc, P P Barbosa MSc, M R Amorim MSc, J Forato BSc, J L Proença-Módena PhD) and Hub of Global Health (J L Proença-Módena), University of Campinas, Campinas, Brazil; MRC Centre for Global Infectious Disease Analysis, Department of Infectious Disease Epidemiology (D S Candido DPhil, L Buss MD, C Whittaker PhD, I M Claro PhD, N Chandradeva MRes, N R Faria PhD) and The Abdul Latif Jameel Institute for Disease and Emergency Analytics (C Whittaker), School of Public Health, Imperial College London, London, UK; Department of Zoology, University of Oxford, Oxford, UK (D S Candido, I M Claro, N R Faria); Instituto de Medicina Tropical (D S Candido, I M Claro, Prof E C Sabino PhD, N R Faria) and Departamento de Moléstias Infecciosas e Parasitárias (Prof E C Sabino), Faculdade de Medicina da Universidade de São Paulo, São Paulo, Brazil; Biodiversity Research Centre, Federal University of Roraima, Boa Vista, Brazil (F Granja); Ministério da Saúde, Departamento de Articulação Estratégica de Vigilância em Saúde, Brasilia, Brazil (R de Jesus PhD, P S Lemos PhD, E L L Araújo PhD); Instituto de Ciências Biológicas, Universidade Federal de Minas Gerais, Belo Horizonte, Brazil (R de Jesus)

## Abstract

**Background:**

Chikungunya virus (CHIKV) is an *Aedes* mosquito-borne virus that has caused large epidemics linked to acute, chronic, and severe clinical outcomes. Currently, Brazil has the highest number of chikungunya cases in the Americas. We aimed to investigate the spatiotemporal dynamics and recurrence pattern of chikungunya in Brazil since its introduction in 2013.

**Methods:**

In this epidemiological study, we used CHIKV genomic sequencing data, CHIKV vector information, and aggregate clinical data on chikungunya cases from Brazil. The genomic data comprised 241 Brazilian CHIKV genome sequences from GenBank (n=180) and the 2022 CHIKV outbreak in Ceará state (n=61). The vector data (Breteau index and House index) were obtained from the Brazilian Ministry of Health for all 184 municipalities in Ceará state and 116 municipalities in Tocantins state in 2022. Epidemiological data on laboratory-confirmed cases of chikungunya between 2013 and 2022 were obtained from the Brazilian Ministry of Health and Laboratory of Public Health of Ceará. We assessed the spatiotemporal dynamics of chikungunya in Brazil via time series, mapping, age–sex distribution, cumulative case-fatality, linear correlation, logistic regression, and phylogenetic analyses.

**Findings:**

Between March 3, 2013, and June 4, 2022, 253 545 laboratory-confirmed chikungunya cases were reported in 3316 (59·5%) of 5570 municipalities, mainly distributed in seven epidemic waves from 2016 to 2022. To date, Ceará in the northeast has been the most affected state, with 77 418 cases during the two largest epidemic waves in 2016 and 2017 and the third wave in 2022. From 2016 to 2022 in Ceará, the odds of being CHIKV-positive were higher in females than in males (odds ratio 0·87, 95% CI 0·85–0·89, p<0·0001), and the cumulative case-fatality ratio was 1·3 deaths per 1000 cases. Chikungunya recurrences in the states of Ceará, Tocantins (recurrence in 2022), and Pernambuco (recurrence in 2021) were limited to municipalities with few or no previously reported cases in the previous epidemic waves. The recurrence of chikungunya in Ceará in 2022 was associated with a new East-Central-South-African lineage. Population density metrics of the main CHIKV vector in Brazil, *Aedes aegypti*, were not correlated spatially with locations of chikungunya recurrence in Ceará and Tocantins.

**Interpretation:**

Spatial heterogeneity of CHIKV spread and population immunity might explain the recurrence pattern of chikungunya in Brazil. These results can be used to inform public health interventions to prevent future chikungunya epidemic waves in urban settings.

**Funding:**

Global Virus Network, Burroughs Wellcome Fund, Wellcome Trust, US National Institutes of Health, São Paulo Research Foundation, Brazil Ministry of Education, UK Medical Research Council, Brazilian National Council for Scientific and Technological Development, and UK Royal Society.

## Introduction

Chikungunya virus (CHIKV) is a major threat to global public health, and is mainly transmitted among humans by *Aedes aegypti* and *Aedes albopictus* mosquitoes.^1^ During the past 20 years, over 10 million cases of chikungunya have been reported in more than 125 countries or territories.^2^ Chikungunya is a disease characterised by acute and chronic signs and symptoms, typically with severe and often chronic arthralgia, and can sometimes lead to some neurological complications and fatal outcomes.^2,3^ 1·3 billion people have been estimated to live in areas at risk of CHIKV transmission.^4^ Modelling suggests that many more parts of the world might become suitable for CHIKV transmission due to climate change increasing the distribution of *Ae aegypti*.^5^ No licensed vaccines or antiviral drugs are available to prevent CHIKV infection or treat chikungunya.

CHIKV is currently classified into West African, East-Central-South-African (ECSA), and Asian genotypes.^1^ The ECSA genotype gave rise to the Indian Ocean lineage (IOL), which has been responsible for epidemics since 2005 in the Indian Ocean islands, south and southeast Asia, and Europe.^1,2^ The expansion of the ECSA-IOL epidemic has been partly attributed to adaptive mutations in the E1 and E2 virus envelope glycoproteins, which facilitated adaptation for *Ae albopictus* infection and transmission.^6,7^ CHIKV infection appears to promote life-long immunity, in which neutralising antibodies prevent disease recurrence and potentially reinfection.^8^ Chikungunya outbreak recurrences in Africa and Asia are often preceded by long periods spanning several years or decades with minimal or no cases. Recurrence can be explained by several factors, including the absence of neutralising antibodies in younger age groups after periods of epidemiological silence.^9,10^ Additionally, recurrences of chikungunya in west Africa have been attributed to enzootic CHIKV periodically causing spillover infections in people who enter or live near forests, causing individual cases and small outbreaks.^10^ The periodicity of these spillover cases appears to be driven by changes in herd immunity among non-human primate enzootic hosts.^11^ However, the dynamics and drivers associated with chikungunya recurrence in urban environments are poorly understood.

In Brazil, where *Ae aegypti* and *Ae albopictus* are widely distributed,^12^ autochthonous chikungunya cases caused by the Asian genotype were first detected in December, 2013, and those caused by the ECSA genotype were first detected in September, 2014.^13^ Subsequent genomic investigations indicated the predominance of the ECSA genotype across Brazil’s five geographic regions (the north, northeast, central-west, southeast, and south). Currently, Brazil has the highest number of chikungunya cases in the Americas. In this study, we contextualise the spread of CHIKV in Brazil from 2013 to 2022 and combine epidemiological, genomic, and vector density population analyses to describe and investigate chikungunya recurrence.

## Methods

### Study design and population

This epidemiological study combined CHIKV genome sequencing data, CHIKV vector information, and aggregate clinical data on chikungunya cases from Brazil. National epidemiological data on laboratory-confirmed cases of chikungunya were obtained from the Brazilian Ministry of Health. This dataset included the aggregate number of cases of chikungunya per epidemiological week from all municipalities of Brazil from epidemiological week 10 (March 3–9) in 2013 to epidemiological week 22 in 2022 (May 29 to June 4). Epidemiological data on laboratory-confirmed cases of chikungunya and dengue in the state of Ceará, from Jan 1, 2015, to May 31, 2022, were obtained from the Central Laboratory of Public Health of Ceará. Individualised and deidentified data were collected on all patients tested for CHIKV and dengue virus (DENV) in Ceará. These data included patient age, sex, and municipality residence, date of symptoms, date of sample collection, diagnosis method, and clinical outcome (mortality). Additionally, for sequencing analysis, we used residual serum samples from patients who tested positive for CHIKV by quantitative RT-PCR (RT-qPCR) between Feb 7 and May 21, 2022, in Ceará (ie, coinciding with the most recent outbreak of chikungunya in 2022). All serum samples were obtained from patients cared for in the public health system, for whom samples were submitted to the Central Laboratory of Public Health of Ceará as part of the surveillance system of Ceará. All study procedures followed the ethical standards of the responsible committee on human experimentation and were approved by the ethics committee of the University of Campinas, Campinas, Brazil (approval number 53910221.0.0000.5404). Individual patient-informed consent was not required for this retrospective study of anonymised samples that were submitted for diagnostic and surveillance testing.

### Procedures

For the patients sampled in our genome sequencing analysis, basic clinical and demographic data were collected from the Brazilian Laboratorial Environment Management System. Anonymised patient information data for all samples used in the current study are provided in appendix 2 (pp 7–8). We extracted viral RNAs from the serum samples, and tested the RNAs by specific real-time RT-qPCR for CHIKV as described previously.^14^ RNA samples positive for CHIKV by RT-qPCR with cycle threshold (Ct) values less than 30 were submitted for CHIKV genome sequencing by a nanopore sequencing approach, as described in appendix 2 (p 1).

We included CHIKV sequences with greater than 85% genome coverage in the final analysis. The new sequences were appended to 180 other Brazilian CHIKV complete coding sequences available in GenBank (from database inception to May 31, 2022; appendix 2 pp 9–14) and classified into genotypes on the basis of the 180 previous sequences. Subsequently, multiple sequence alignment of the 241 sequences was done in MAFFT (version 7.450),^15^ and manual adjustment was conducted with Geneious Prime (version 2020.2.3). The dataset was screened for recombination events with use of all available methods (BOOTSCAN, GENECONV, MAXCHI, CHIMAERA, SISCAN, 3SEQ, VisRD, and BURT) in Recombination Detection Program (version 4).^16^ No evidence of recombination was found. We subsequently performed maximum likelihood phylogenetics using IQ-TREE (version 2) under a GTR + I + γ model as determined by ModelFinder, where GTR (ie, General Time Reversible) is the variable base frequencies (symmetrical substitution matrix), I is the proportion of invariable sites, and γ is the γ-distributed rate variation among sites.^17,18^ Statistical support for nodes of the maximum likelihood phylogeny was assessed by an ultrafast-bootstrap approach with 1000 replicates. We then regressed root-to-tip genetic divergence against sampling dates to investigate the temporal signal and identify sequences with low data quality in our dataset, caused by assembling issues, sample contamination, data annotation errors, sequencing, and alignment errors. No obvious outliers were identified in this step. Dated phylogenetic trees were estimated with use of BEAST (version 1.10.4) under a GTR + I + γ model, strict molecular clock model, and a Skygrid tree prior, and with use of BEAGLE (version 3.1.0) to enhance computation speed. Markov chain Monte Carlo chains were run for over 50 million generations and sampled every 1000 steps, with convergence assessed with Tracer (version 1.7).^19^ Maximum clade credibility summary trees were summarised with TreeAnnotator (version 1.10).

We also analysed Breteau index and House index for all 184 municipalities in Ceará state and for 116 of 139 municipalities in Tocantins state, which were assessed between Jan 3 and Feb 21, 2022, as part of the *Ae aegypti* Infestation Index Rapid Survey (applied by Brazilian municipalities since 2003).^20^ The indices data were provided by the Brazilian Ministry of Health. The Breteau index is the number of water containers containing *Ae aegypti* larvae per 100 houses, and the House index is the percentage of houses with infested containers. Additionally, we used digital surveillance data via the Google Trends tool to compile the monthly fraction of online searches for the term “chikungunya” that originated from Brazil from Jan 1, 2013, to May 31, 2022, and plotted these data as a time series.

### Statistical analysis

All analyses were done in R studio (version 1.3.1073). Incidence of chikungunya was calculated per 100 000 inhabitants on the basis of the estimated populations of Brazilian states from 2013 to 2022, as reported by the Brazilian Institute of Geography and Statistics. Based on national data, epidemiological dynamics in terms of chikungunya case numbers and incidence were presented annually and by state to identify epidemic waves, defining the start and end of waves by upward or downward periods in case numbers that were substantial through being sustained over time.^21^ In addition, using data from the Central Laboratory of Public Health of Ceará, we analysed the temporal distribution of chikungunya case numbers, incidence, and deaths at the state level and across all 184 Ceará state municipalities, with incidence during epidemic waves stratified by sex (male and female) and 10-year age group. Data were also summarised for the states of Pernambuco and Tocantins. Logistic regression was used to calculate odds ratios (ORs) with 95% CIs to assess statistical differences in the probability of chikungunya (in all individuals tested for CHIKV in Ceará) or probability of death (among all individuals who died and had tested positive for CHIKV in Ceará) by sex and age group (<18 years, 18–39 years, 40–54 years, 55–74 years, and ≥75 years), incorporating both variables as covariates. The correlation between laboratory-confirmed chikungunya-related deaths per month and chikungunya cases per month in Ceará was determined by Pearson’s correlation coefficients. Cumulative case-fatality ratio by sex and 10-year age group was also calculated. Case data, correlation analyses, and case-fatality ratio are presented for dengue cases in Ceará for comparison.

We assessed correlations between measures of surveillance of chikungunya. We evaluated the correlation between Google Trends data for chikungunya and laboratory-confirmed chikungunya cases per Brazilian federal units using Spearman’s rank correlation test. Correlation coefficients of Breteau and House indices per chikungunya incidence in 2022 in Ceará state municipalities and Tocantins state municipalities were also calculated with Spearman’s rank correlation test.

Differences by sex and age group (<18 years, 18–64 years, and ≥65 years) in Ct values for chikungunya cases in Ceará in 2022 (n=638), and in the interval between symptom onset and sample collection, were calculated by one-way ANOVA with Tukey’s honestly significant difference test.

Figures and maps were coloured according to recommendations for scientifically derived colour maps.^22^ In all tests, significance was defined as a p value of less than 0·05.

### Role of the funding source

The funders of the study had no role in study design, data collection, data analysis, data interpretation, or writing of the report.

## Results

**Figure 1:**
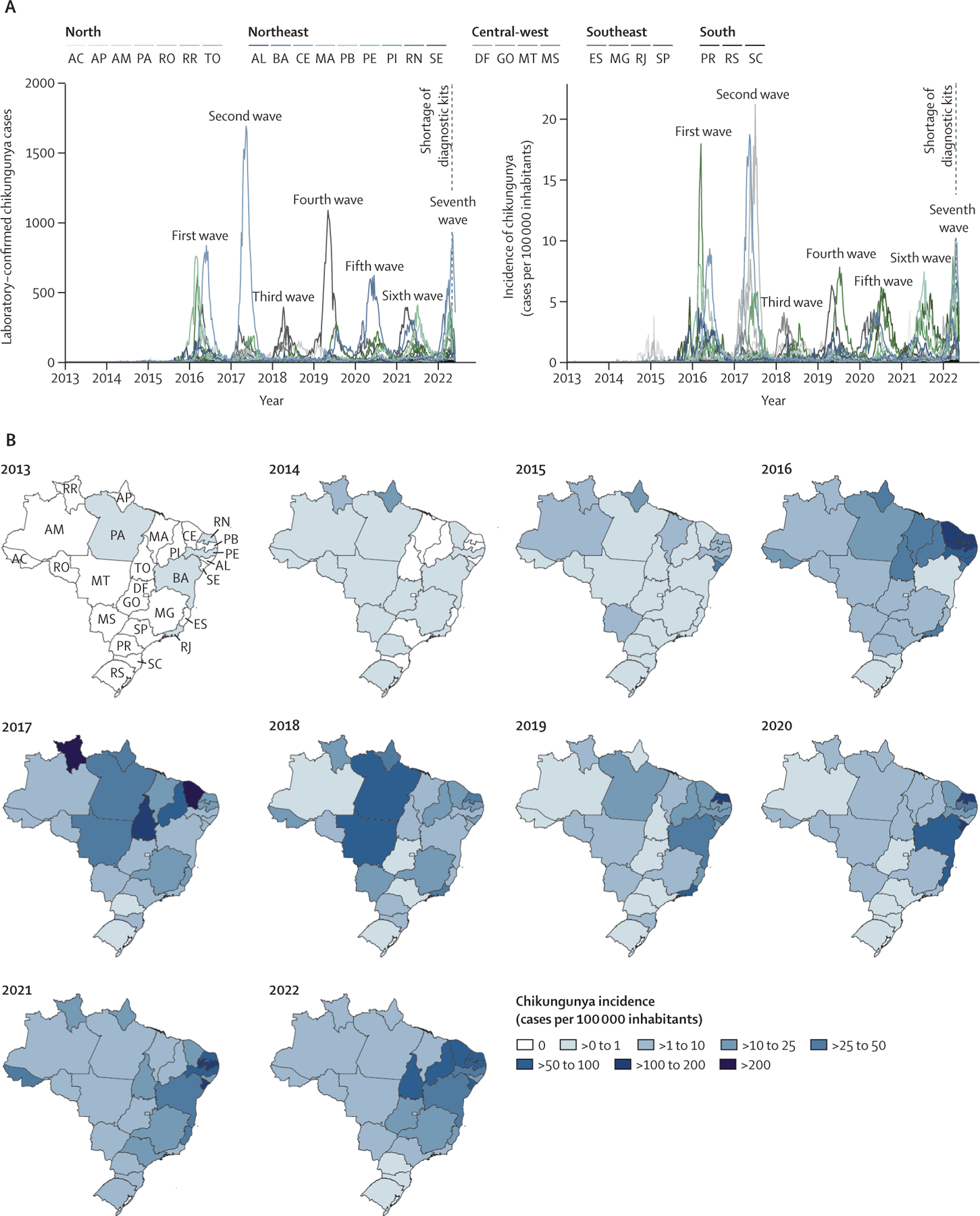
Spatiotemporal dynamics of chikungunya between 2013 and 2022 in Brazil (A) Number of laboratory-confirmed chikungunya cases and incidence according to laboratory-confirmed chikungunya cases per epidemiological week in all 26 Brazilian States and the Federal District, from epidemiological week 10 of 2013 (March 3–9) to epidemiological week 22 of 2022 (May 29 to June 4). (B) Maps coloured according to the incidence of laboratory-confirmed chikungunya cases per state. The map for 2022 is limited to epidemiological weeks 1–22. AC=Acre. AL=Alagoas. AM=Amazonas. AP=Amapá. BA=Bahia. CE=Ceará. ES=Espírito Santo. DF=Distrito Federal (Federal District). GO=Goiás. MA=Maranhão. MG=Minas Gerais. MS=Mato Grosso do Sul. MT=Mato Grosso. PA=Pará. PB=Paraíba. PE=Pernambuco. PI=Piauí. PR=Paraná. RJ=Rio de Janeiro. RN=Rio Grande do Norte. RO=Rondônia. RR=Roraima. RS=Rio Grande do Sul. SC=Santa Catarina. SE=Sergipe. SP=São Paulo. TO=Tocantins.

Between March 3, 2013, and June 4, 2022, 253 545 laboratory-confirmed chikungunya cases were reported to the Brazilian Ministry of Health across 3316 (59·5%) of 5570 municipalities in all 26 states and the Federal District. Chikungunya cases were mainly distributed across seven large epidemic waves between 2016 and 2022, which resulted in 24 097 to 44 604 confirmed cases annually (figure 1A, appendix 2 p 8). The epidemic peak of cases in most affected Brazilian states varied annually between February and July (appendix 2 p 8). Google searches of the search term “chikungunya” in Brazil captured these seven large epidemic waves, with a high correlation (Spearman’s *r*=0·74, p<0·0001) between Google Trends data and the number of chikungunya cases in the states most affected (appendix 2 p 2). Since early May 2022 (epidemiological week 18; May 1–7), we observed a decrease of 75·1% in reported chikungunya cases (epidemiological week 18, n=2493 cases and epidemiological week 22, n=620 cases), probably due to a shortage of diagnostic kits for CHIKV, DENV, and Zika virus (ZIKV) in Brazil^23^ (figure 1A). Northeast Brazil was the region most affected by chikungunya, which had 160 909 (63·5%) of all 253 545 reported cases between 2013 and 2022 (figure 1A). Ceará had the highest number of cases (n=45 417) and a cumulative incidence of 501·4 cases per 100 000 inhabitants from 2013 to 2022 (figure 1A, 1B).

**Figure 2:**
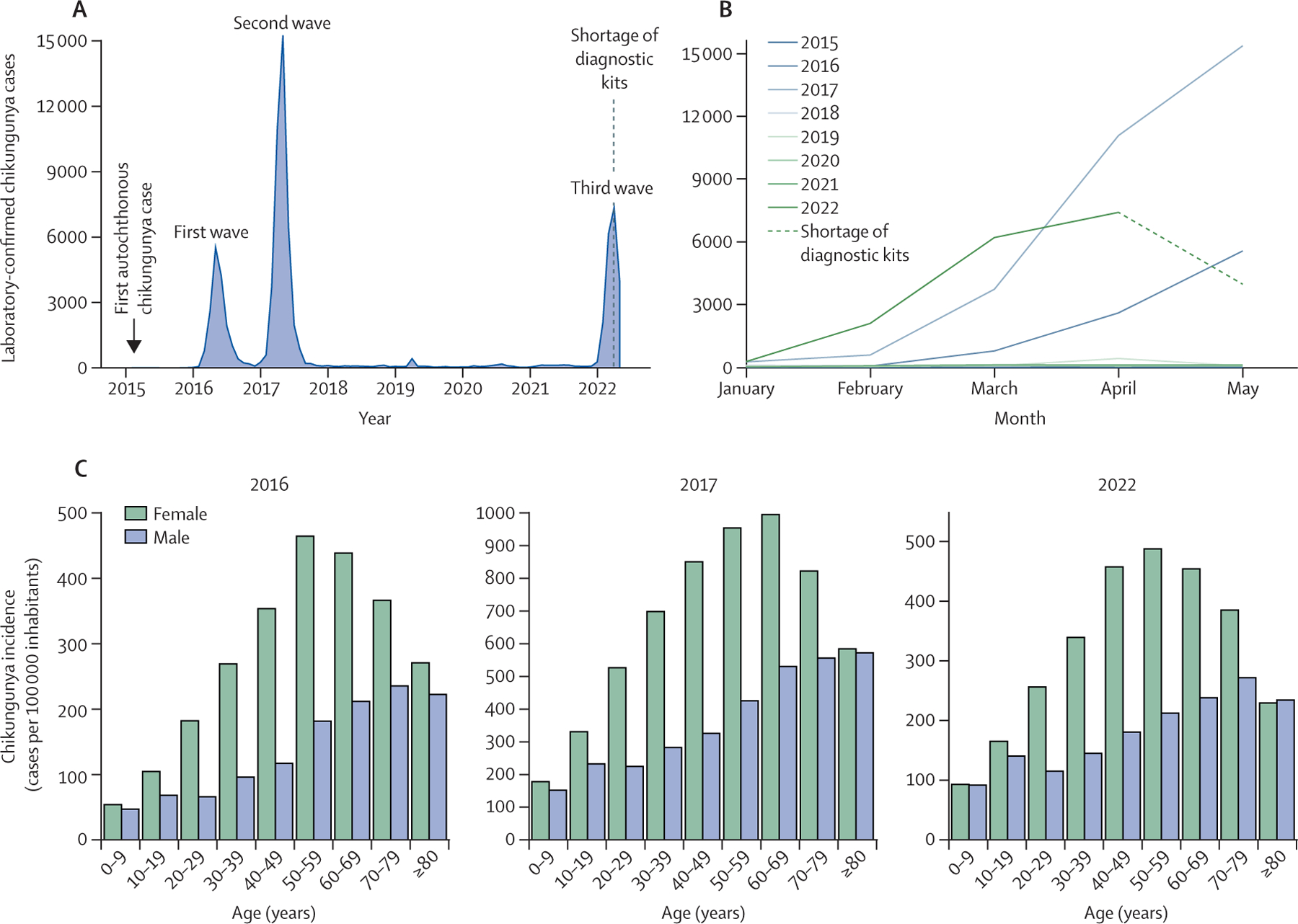
Chikungunya waves in Ceará state, Brazil (A) Number of laboratory-confirmed chikungunya cases per month from Jan 1, 2015, to May 31, 2022. (B) Number of laboratory-confirmed chikungunya cases for the first five months of each year (2015–22); datapoints correspond to total cases per month. (C) Chikungunya incidence based on age–sex distribution of epidemic waves in 2022, 2017, and 2016.

We analysed the individualised data of all patients tested for CHIKV (n=146 887) in the Central Laboratory of Public Health of Ceará from Jan 1, 2015, to May 31, 2022. Ceará had three chikungunya waves since the first autochthonous case was reported on March 6, 2015, in Fortaleza, the state’s capital and most populous city (figure 2A). In the first wave in Ceará, 17 012 chikungunya cases were reported overall, and the wave reached an epidemic peak (5523 [32·5%] cases) in May, 2016. In the second wave, 40 596 cases were reported overall, and the wave reached a peak (15 257 [30·2%] cases) in May, 2017. After 4 years (December, 2017, to December, 2021) of low chikungunya incidence (≤1 case per 100 000 inhabitants), the disease reoccurred in a third epidemic in 2022, with 19 810 confirmed cases overall up to the last recorded timepoint in this study (May 31, 2022). Comparing the first 5 months of each epidemic year in Ceará, the third wave caused 2·2-times more cases than the first wave (8956 cases from January to May, 2016), but 1·6-times fewer cases than the second wave (31 802 cases from January to May, 2017; figure 2B). In addition, our analysis of the age–sex structure of chikungunya cases in the three epidemic waves showed a higher incidence (2·2 to 3·0-times higher) in women aged 20–59 years than in men of the same ages (figure 2C). From 2016 to 2022, the sex disparity was significant, with males having consistently lower odds of being CHIKV-positive than females (OR 0·87, 95% CI 0·85–0·89, p<0·0001; appendix 2 p 9). Adult age groups also had significantly higher odds of being CHIKV-positive than young patients (aged <18 years; appendix 2 p 9). Based on analysis of a set of RT-qPCR-confirmed CHIKV-positive cases collected during the third wave (n=638), no significant difference was observed in median Ct values between sexes (appendix 2 p 3). In addition, we found that young patients (<18 years) had significantly lower median Ct values than the older age groups (18–64 years and ≥65 years), indicating that young patients had higher serum concentrations of viral nucleic acid than adults. Patients younger than 18 years and those aged 18–64 years had a median interval between symptom onset and sample collection of 2 days, compared with 3 days in older adults (≥65 years), although only the difference between the two adult age groups (18–64 years *vs* ≥65 years) was significant (appendix 2 p 3).

**Figure 3:**
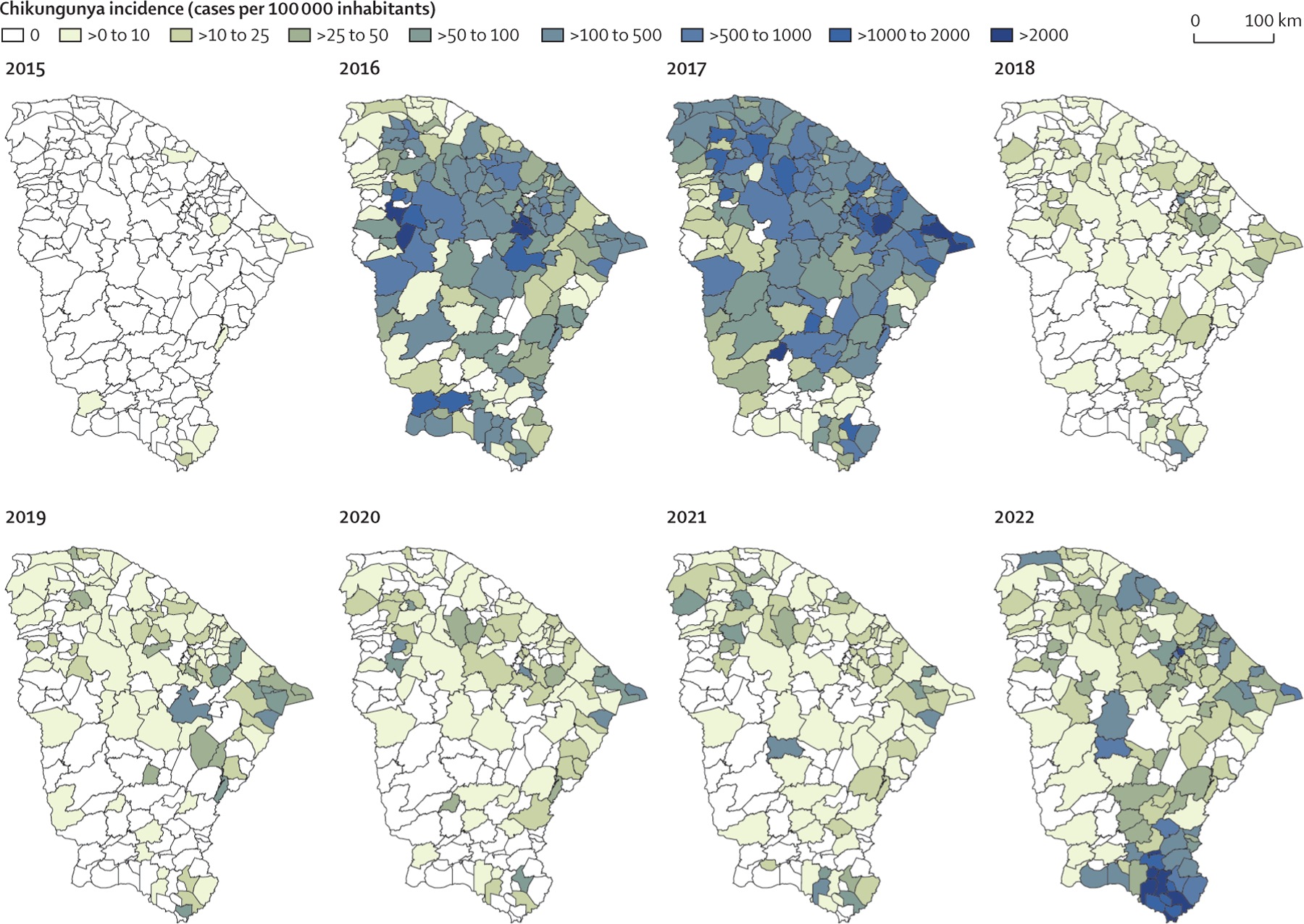
Spatiotemporal distribution of chikungunya recurrence in Ceará state, Brazil Spatiotemporal distribution of annual chikungunya incidence based on laboratory-confirmed chikungunya cases per municipality (n=184 municipalities) in Ceará from 2015 to 2022. Chikungunya incidence in 2022 includes data up to May 31.

We subsequently analysed the spatial distribution and incidence of chikungunya cases across the 184 municipalities in all seven mesoregions of Ceará from 2015 to 2022. We observed that chikungunya recurrence in 2022 predominantly affected municipalities in the south of Ceará (figure 3). The third epidemic wave occurred in a small number of municipalities (n=37; cumulative incidence since the start of the wave of ≥100 chikungunya cases per 100 000 inhabitants) compared with 100 municipalities (≥100 chikungunya cases per 100 000 inhabitants) in the first and second waves. In addition, five of seven of the Ceará mesoregions reported more than 800 cases and an incidence of more than 100 cases per 100 000 inhabitants of Ceará per month during the first or second chikungunya waves. The two regions with lower incidence were Centro-Sul Cearense and Sul Cearense. Conversely, Sul Cearense was the most affected mesoregion by the 2022 epidemic wave (appendix 2 p 4). Comparing chikungunya incidence caused by the first and second waves (2016 and 2017) with incidence in the third wave (2022), we found that municipalities most affected by the recurrence of chikungunya were less affected during the first two epidemics. We found a similar pattern in the states of Pernambuco (chikungunya recurrence in 2021) and Tocantins (chikungunya recurrence in 2022; appendix 2 p 4). These data show that chikungunya has recurred primarily in regions and municipalities that were less affected during previous epidemic waves in Brazil.

**Figure 4:**
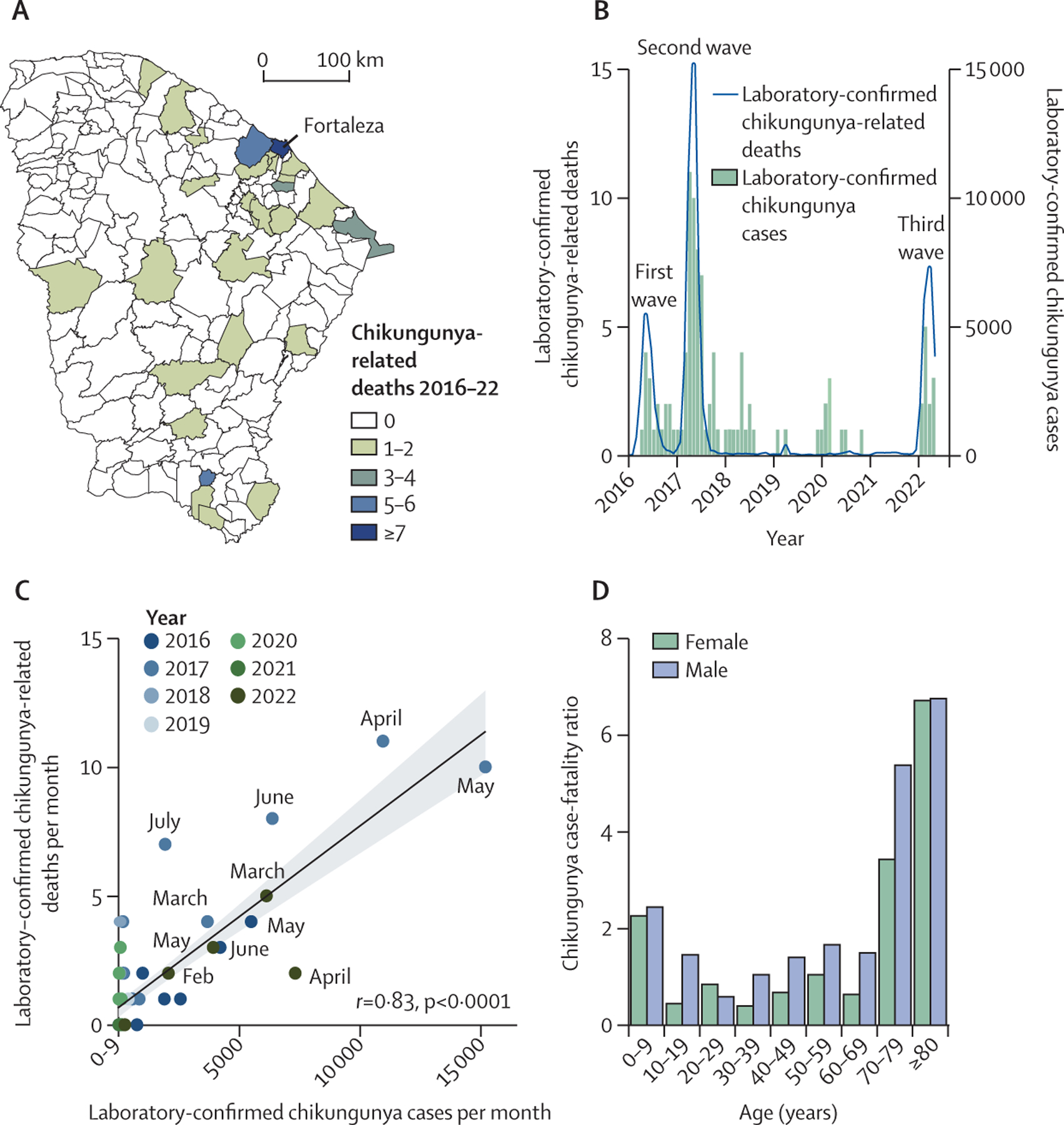
Chikungunya-related deaths in Ceará state, Brazil (A) Spatial distribution of laboratory-confirmed chikungunya-related deaths per municipality (n=184 municipalities) in Ceará from 2016 to 2022. (B) Number of laboratory-confirmed chikungunya-related deaths and cases per month from January, 2016 to May, 2022. (C) Pearson’s correlation between laboratory-confirmed chikungunya-related deaths per month and laboratory-confirmed chikungunya cases per month from January, 2016, to May, 2022, with key months labelled. (D) The cumulative chikungunya case fatality ratio by age and sex from January, 2016, to May, 2022.

From Feb 10, 2016, to May 31, 2022, 103 deaths due to laboratory-confirmed chikungunya were reported across 28 municipalities in Ceará, with 55 (53·4%) of 103 deaths reported in Fortaleza (figure 4A). The peaks in deaths overlapped with the peaks in the number of chikungunya cases during the three chikungunya waves (figure 4B). The cumulative case-fatality ratio from 2016 to 2022 was 1·3 deaths per 1000 cases. In addition, we found a positive correlation between deaths and cases per month (Pearson’s *r*=0·83, p<0·0001; figure 4C). Across the 184 municipalities of Ceará, the cumulative proportion of inhabitants with laboratory-confirmed chikungunya reached 6·9% (ie, Pena Forte in the third epidemic wave). The odds of chikungunya-related death did not appear to differ significantly between females and males. Compared with individuals younger than 18 years, the odds of chikungunya-related death were significantly increased in those aged 55–74 years (OR 2·28, 95% CI 1·11–4·68, p=0·025) and those aged 75 years or older (4·01, 1·83–8·78, p=0·0005; appendix 2 p 9). Additionally, we identified a U-shaped pattern in age-associated mortality (figure 4D). In comparison, dengue caused 72 deaths in 29 municipalities in Ceará during the same period, and most cases (33 [45·8%] of 72) were reported in Fortaleza. A negligible correlation was observed between dengue-related laboratory-confirmed deaths and monthly cases (appendix 2 p 5). The cumulative case-fatality ratio for dengue was estimated at 1·1 deaths per 1000 laboratory-confirmed cases. In descriptive terms, dengue-related deaths were reported in more males than females (male-to-female ratio of 1·1; appendix 2 p 5).

**Figure 5:**
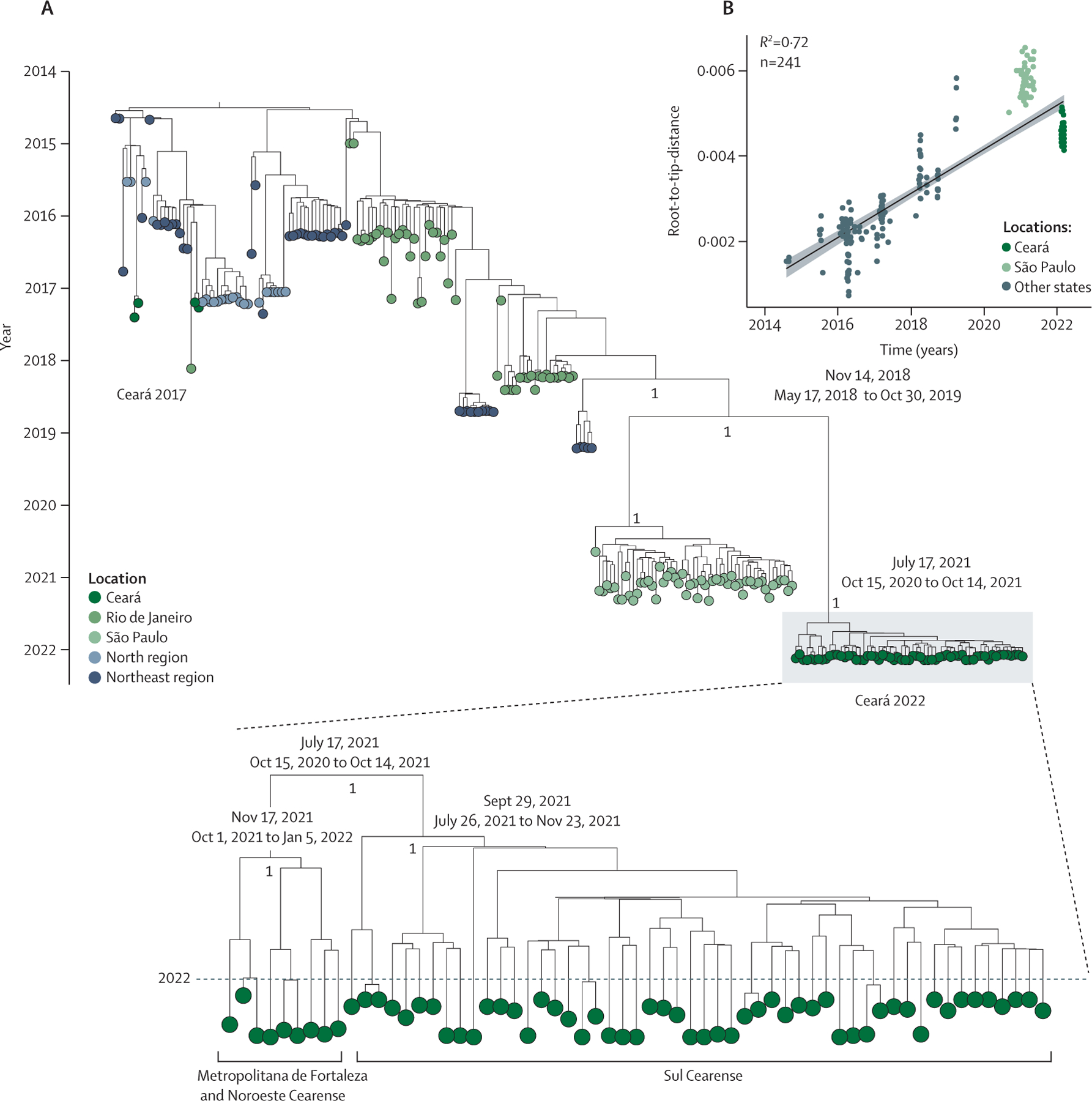
Phylogenetic analysis of the ECSA genotype of CHIKV in Brazil (A) Maximum clade credibility tree of 241 CHIKV genomes from the ECSA genotype, including 61 new CHIKV genomes (Ceará 2022, magnified section) from Ceará state generated in this study. Tips are coloured according to the source region or state of each sample. A strict molecular clock approach was used for generating the time-rooted tree. Posterior probability scores are shown next to key well supported nodes. Dates at key nodes are the estimated date of divergence from a common ancestor, with Bayesian credible intervals. (B) Regression of sequence sampling dates against root-to-tip genetic distances in a maximum likelihood phylogeny of the CHIKV-ECSA genotype in Brazil. Sequences are coloured according to source locations. ECSA=East-Central-South-African. CHIKV=chikungunya virus.

We investigated the genetic diversity of CHIKV in Ceará in 2022. We sequenced 61 genomes from patients who tested positive for CHIKV during the recent outbreak between Feb 7 and March 9, 2022. The samples were from three mesoregions (Metropolitana de Fortaleza, n=3 genomes; Noroeste Cearense, n=6; and Sul Cearense, n=52; figure 5A, appendix 2 pp 7–8). All genomes had at least 85% coverage and a mean depth of coverage of at least 20 ×. A regression of genetic divergence from root to tip against sampling dates confirmed a strong temporal signal in our genomic dataset (figure 5B). We estimated a timescale for the evolution of the CHIKV clade in 2022 using a strict molecular clock model. Phylogenetic analysis revealed that an introduction of a new CHIKV-ECSA lineage caused the third epidemic wave in Ceará (figure 5A). This new lineage was most closely related to CHIKV lineages circulating recently in São Paulo state, and the most recent common ancestor of the CHIKV-ECSA lineage associated with chikungunya recurrence in Ceará was estimated on or around July 17, 2021 (95% Bayesian credible interval Oct 15, 2020, to Oct 14, 2021). We did not find previously described mutations^6,7,24^ associated with enhanced transmission potential for *Ae albopictus* mosquitoes (eg, E1–A226V and E1–T98A) in the CHIKV strains circulating in 2022, consistent with a scarcity of incrimination of this species in transmission in the Americas.^25^ Additionally, we did not find epistatic interactions that control penetrance of the E1–A226V mutation (ie, E1–K211T and E1–98T), which are associated with adaptation to a new vector, and thus can restrict epidemic emergence.^24^

We assessed whether *Ae aegypti* population density correlated with the increase in CHIKV transmission in 2022. For this analysis, we evaluated Breteau index and House index for all Ceará state municipalities and for 116 (83·5%) of 139 Tocantins state municipalities from Jan 3, to Feb 21, 2022. In Ceará, we found that 72 (39·**1**%) of 184 municipalities had a Breteau index or House index higher than 1, which triggers a risk alert for *Ae aegypti-*borne virus outbreaks according to the National Dengue Control Programme in Brazil^26^ (appendix 2 p 6). By contrast, we identified that 99 (85·3%) of 116 municipalities in Tocantins had a Breteau index or House index higher than 1 (appendix 2 p 6). However, no correlation was observed between *Ae aegypti* indices and chikungunya incidence in Ceará or Tocantins municipalities in 2022 (appendix 2 p 4). Additionally, we did not find a correlation between *Ae aegypti* indices (Breteau index or House index) and dengue incidence in Ceará in 2022 (appendix 2 p 6). We did not evaluate the correlation between chikungunya recurrence and *Ae aegypti* indices in Pernambuco due to the absence of vector data for the recurrence period in 2021. These data suggest that vector density, as measured by traditional indices, was not a major driver of the recurrence of chikungunya in Ceará and Tocantins.

## Discussion

In this epidemiological study, we have provided a comprehensive assessment of chikungunya epidemics and recurrence in Brazil. We have described and contextualised the spatial and temporal dynamics of seven large chikungunya epidemic waves in Brazil between 2013 and 2022, and explored recurrence of chikungunya after a 4-year interval in three Brazilian states. We found that Ceará was the most affected Brazilian state, where chikungunya recurrence in 2022 resulted from a new lineage of CHIKV-ECSA introduced in mid-2021, which was distinct to previous lineages circulating in this state.^3^ However, this new viral lineage does not alone explain the observed recurrence, given that previous CHIKV infection produces a robust humoral response that prevents reinfection, even against other genotypes.^8,27^ Instead our analysis suggests that the spatial heterogeneity of CHIKV spread during the first waves might at least partially explain recent chikungunya recurrence. We found that municipalities in the states of Ceará, Pernambuco, and Tocantins that experienced chikungunya recurrence were affected less or completely unaffected by previous waves. By contrast, regions largely affected in previous waves had low incidence or no recorded chikungunya cases during the recurrence of chikungunya in 2022. Therefore, the populations in municipalities most affected by early waves of chikungunya might have had some level of immune protection against disease or transmission that temporarily prevented the recurrence of large chikungunya outbreaks. By contrast, the populations from municipalities who were less exposed to early waves of CHIKV remained susceptible.

Our results show that chikungunya affected females more than males, consistent with previous findings that suggested females are 1·5-times more likely to become infected with CHIKV than males.^28^ We also estimated a case-fatality ratio of chikungunya in Ceará of 1·3 deaths per 1000 cases. This estimate is similar to the ratio previously reported for Réunion Island in the Indian Ocean in 2005–06 (1 death per 1000 chikungunya cases) with a high sensitivity surveillance system,^29^ which was also consistent with iterative external studies and serosurveys.^30^ Collectively, our results indicate that chikungunya outbreaks can be rapid and result in a large number of cases, and a case-fatality ratio similar to that seen in dengue epidemics (1·1 deaths per 1000 laboratory-confirmed cases in our analysis). Therefore, mechanisms and potential biomarkers associated with severe chikungunya outcomes need to be investigated to support the development of antiviral therapies, improve clinical management, and reduce chikungunya burden.

We found that the peaks of chikungunya cases in epidemic waves occurred mainly between February and June in Brazil, coinciding with the rainy season and increased temperatures. These wet and warm periods have been described as crucial drivers of the magnitude and seasonality of mosquito-borne virus transmission, by affecting mosquito reproduction, survival, biting rates, and the adult vector population density.^31^ Consequently, these conditions can increase the risk and dynamics of arbovirus transmission, such as for CHIKV and DENV. However, our results also indicate that CHIKV circulation is stable during other parts of the year. Overwintering of vectors is probably explained by variable rainy and warm seasons throughout Brazil, combined with the absence of cold winters and high vector endemicity in all states. Additionally, in our analysis, *Ae aegypti* population density metrics were not correlated spatially with locations of chikungunya recurrence in Ceará and Tocantins. These findings highlight that vector population density thresholds (eg, House index and Breteau index) need to be improved by incorporating herd immunity data to improve predictions of outbreaks transmitted by *Ae aegypti* and *Ae albopictus*. Robust modelling that accounts for mosquito population dynamics, climate data, immunity to CHIKV, and the distribution of *Ae aegypti* control interventions across the country could help to quantify chikungunya burden and evaluate the effect of vector density on chikungunya recurrence.

There are several limitations to our study. First, the absence of CHIKV genomic sequences from other Brazilian states prevented us from accurately concluding on the date of introduction and geographical origins of the new lineage in Ceará. The implementation of screening of blood donors combined with CHIKV genome sequencing could provide unique data to understand the epidemiological and evolutionary dynamics of CHIKV in Brazil. Second, the absence of CHIKV seroprevalence studies at the state and national levels in Brazil limits estimates of susceptibility and herd immunity in the population. For example, some CHIKV-endemic countries, such as India, have an overall prevalence of IgG antibodies against CHIKV of 18%.^32^ By contrast, our data show that no more than 7% of the populations of municipalities most affected received a laboratory diagnosis of chikungunya in the three waves in Ceará. Several factors could explain these discrepancies, such as oligosymptomatic and asymptomatic cases (up to 80% of cases),^33^ challenges of the syndromic surveillance systems due to the co-circulation of ZIKV and DENV, which cause similar symptoms to chikungunya,^34^ and variable health-care-seeking behaviours. Third, the shortage of diagnostic kits for arboviruses during the epidemic (due to case numbers exceeding expected demand when compared with the same period in 2021^23^) might underestimate the chikungunya burden in 2022 because endemic DENV and ZIKV infections are easily confused. Fourth, information was absent on individual-level factors related to socioeconomic status and deprivation that can be important determinants of arboviral risk,^35^ which should be explored in the context of Brazilian CHIKV outbreaks in future research. Finally, comparisons with the epidemiology of DENV and ZIKV in Brazil are needed to improve understanding of their apparent differences in outbreak dynamics.

In conclusion, our findings provide important context about the dynamics and drivers of chikungunya in Brazil, and might inform future studies and public health policy on strategies to mitigate the effects of new epidemic waves in urban settings and immunisation programmes. For example, subsequent modelling studies based on the present data could predict potential hotspots for new chikungunya epidemic waves. Such studies could be followed by optimised and focused public health countermeasures such as mosquito control in identified hotspots, and could inform further vaccine prioritisation strategies. Additionally, chikungunya should be considered as a possible death diagnosis in endemic countries to improve understanding of the burden of fatal disease.

## Data sharing

All statistical computing analyses were conducted with use of the R project. R packages used in this study for time series and age–sex distribution analysis were tidyverse, ggpubr, and scico, and spatiotemporal analysis was performed with raster, tmap, sf, and scico. No custom code was developed. New chikungunya virus sequences have been deposited in GenBank with accession numbers OP964932 to OP964992. All clinical and laboratory datasets aggregated and presented in the present study are available on GitHub (https://github.com/wmarciel/Chikungunya-in-Brazil-2013-2022.git). The deidentified individualised data provided by the Brazilian Ministry of Health and the Central Laboratory of Public Health of Ceará can be made available for research purposes; any future research should be approved by a committee on human experimentation. The data can be provided upon request to the corresponding author. The Breteau index and House index data were obtained from the Brazilian Ministry of Health through the Right to Information Law (Law 12.527, Brazil) under process number 25072.015694/2022–81.

## Supplementary Material

1

2

## Research in context

### Evidence before this study

Chikungunya virus (CHIKV) is a mosquito-borne virus that has spread and caused more than 10 million cases in over 125 countries or territories in the past 20 years. Since the introduction of CHIKV in the Americas in December, 2013, Brazil has reported more than 1 million chikungunya cases, the highest number in the Americas. We searched PubMed without language restrictions from database inception up to May 2, 2022, for studies published with the terms “chikungunya recurrence” or “chikungunya re-emergence” or “chikungunya emergence”. We found evidence of CHIKV re-emergence or reoccurrence in Asia and Africa in young, naive populations after several years of low incidence or no infections; however, most of the studies on chikungunya re-emergence or recurrence were limited to rural areas, and there appears to be long periods without reported cases (up to decades) between outbreaks. To our knowledge, information is scarce on the recurrence pattern of chikungunya over short time periods and in urban environments, such as in the Americas.

### Added value of this study

We report the spatial and temporal dynamics of seven epidemic waves of chikungunya across Brazil, and show that the recurrence pattern of chikungunya in urban environments is due to spatial heterogeneity in viral spread, with regions that experienced previous epidemic waves and associated herd immunity showing reduced recurrence of chikungunya. However, recurrence of chikungunya was not correlated with high vector population density. In addition, we found that females were more likely than males to contract CHIKV, and estimated a chikungunya case-fatality ratio of 1·3 deaths per 1000 confirmed cases, which is higher than for other endemic arboviruses in tropical countries, such as dengue virus and Zika virus.

### Implications of all the available evidence

Our findings suggest that chikungunya epidemics will continue to occur, causing major epidemic waves with thousands of cases and deaths due to the geographical heterogeneity associated with CHIKV spread or recurrence. These results will be useful in informing public health interventions to anticipate and prevent future chikungunya epidemic waves in urban settings.
