# Supplementary material for: Spatiotemporal dynamics and recurrence of chikungunya virus in Brazil: an epidemiological study
Source: Lancet Microbe. Author manuscript; Available in PMC 2023 Jun 20. (PMC10281060; doi:10.1016/S2666-5247(23)00033-2)
Supplement: 1 [file NIHMS1897922-supplement-1.pdf]

THE LANCET
Microbe

Supplementary appendix 1

This translation in Portuguese was submitted by the authors and we reproduce it as supplied. It has not been peer reviewed. The Lancet's editorial processes have only been applied to the original in English, which should serve as reference for this manuscript.

Esta tradução em português foi submetida pelos autores e nós não fizemos quaisquer alterações. Esta versão não foi revista por pares. O processo editorial do The Lancet só foi aplicado à versão original em inglês, que deve servir como referência para este artigo.

Supplement to: de Souza WM, de Lima STS, Simões MelloLM, et al. Spatiotemporal dynamics and recurrence of chikungunya virus in Brazil: an epidemiological study. *Lancet Microbe* 2023; published online April 6. https://doi.org/10.1016/S2666-5247(23)00033-2.

Dinâmica espaço-temporal e recorrência de chikungunya no Brasil: um estudo epidemiológico

Resumo

Introdução: O vírus chikungunya (CHIKV) é transmitido pelos mosquitos do gênero *Aedes* que têm causado epidemias explosivas ligadas a desfechos clínicos agudos, crônicos e graves. Atualmente, o Brasil tem o maior número de casos de chikungunya nas Américas. Este estudo teve como objetivo investigar a dinâmica espaço-temporal e o padrão de recorrência da chikungunya no Brasil desde a sua introdução em 2013

Métodos: Neste estudo epidemiológico, usamos dados de sequenciamento genômico de CHIKV, informações do vetor CHIKV e dados clínicos agregados de casos de chikungunya no Brasil. Os dados genômicos compreendem 241 genomas brasileiros de CHIKV disponíveis no GenBank (n=180) e genomas provenientes do surto de CHIKV 2022 no Ceará (n=61). Os dados vectoriais (índice de Breteau e Predial) foram obtidos com o Ministério da Saúde do Brasil para todos os 184 municípios do Ceará e 116 municípios do Tocantins em 2022. Dados epidemiológicos dos casos confirmados laboratorialmente de chikungunya entre 2013 e 2022 foram obtidos do Ministério da Saúde do Brasil e do Laboratório de Saúde Pública do Ceará. Avaliamos a dinâmica espaço-temporal da chikungunya no Brasil por meio de séries temporais, mapeamento, distribuição idade-sexo, letalidade cumulativa, correlação linear, regressão logística e análises filogenéticas.

Resultados: Entre 3 de março de 2013 e 4 de junho de 2022, foram notificados 253.545 casos de chikungunya confirmados em laboratório em 3.316 (59,5%) dos 5.570 municípios, distribuídos principalmente em sete ondas epidêmicas de 2016 a 2022. Até o momento, o Ceará, no Nordeste, foi o estado mais afetado, com 77.418 casos durante as duas maiores ondas epidêmicas em 2016 e 2017 e o terceira onda em 2022. De 2016 a 2022 no Ceará, a chance de receber diagnóstico CHIKV positivo foi maior no sexo feminino do que no homens (razão de probabilidade de 0,87, intervalo de confiança 95% de 0,85–0,89, p<0,0001), e a taxa cumulativa de letalidade foi de 1,3 mortes por 1000 casos de chikungunya. A recorrência de chikungunya nos estados do Ceará, Tocantins (recorrência em 2022) e Pernambuco (recorrência em 2021) foram limitados a municípios com poucos ou nenhum caso notificado anteriormente nas ondas epidemia prévias. A recorrência da chikungunya no Ceará em 2022 foi associada a uma nova linhagem do genótipo *east-central-south-African*. Métricas de densidade populacional do principal vetor do CHIKV no Brasil, *Aedes aegypti*, não foram correlacionados espacialmente com locais de recorrência de chikungunya no Ceará e Tocantins.

Interpretação: A heterogeneidade espacial da disseminação do CHIKV e a imunidade da população podem explicar o padrão de recorrência da chikungunya no Brasil. Esses resultados podem ser usados para informar intervenções de saúde pública para prevenir futuras ondas epidêmicas de chikungunya em ambientes urbanos.

Financiamento: Global Virus Network, Burroughs Wellcome Fund, Wellcome Trust, National Institutes of Health, Fundação de Apoio à Pesquisa do Estado de São Paulo, Ministério da Ciência do Brasil, Medical Research Council, Conselho Nacional de Desenvolvimento Científico e Tecnológico, e UK Royal Society.
