# Supplementary material for: Spatiotemporal dynamics and recurrence of chikungunya virus in Brazil: an epidemiological study
Source: Lancet Microbe. Author manuscript; Available in PMC 2023 Jun 20. (PMC10281060; doi:10.1016/S2666-5247(23)00033-2)
Supplement: 2 [file NIHMS1897922-supplement-2.pdf]

THE LANCET
Microbe

Supplementary appendix 2

This appendix formed part of the original submission and has been peer reviewed. We post it as supplied by the authors.

Supplement to: de Souza WM, de Lima STS, Simões MelloLM, et al. Spatiotemporal dynamics and recurrence of chikungunya virus in Brazil: an epidemiological study. *Lancet Microbe* 2023; published online April 6. https://doi.org/10.1016/S2666-5247(23)00033-2.

Supplementary appendix

Supplementary Information: Materials and Methods

Definition of cases

Laboratory-confirmed cases of chikungunya or dengue fever were defined as a patient with one positive laboratory result for CHIKV or DENV either by reverse-transcription quantitative polymerase chain reaction (RT-qPCR), immunoglobulin M (IgM) detection, immunoglobulin G (IgG), viral isolation (e.g., C6/36 cells), and/or non-structural protein 1 (NS1) antigen for DENV. No clinical epidemiological cases were included in this study.

Chikungunya virus genome sequencing and assembly genome

Positive RNA samples by RT-qPCR with cycle threshold (Ct) values <30 were submitted for CHIKV genome sequencing using a targeted multiplex PCR scheme and the MinION platform (Oxford Nanopore Technologies, UK), as described elsewhere (1). PCR products were cleaned using AmpureXP purification beads (Beckman Coulter, UK) and quantified using fluorimetry with the Qubit dsDNA High Sensitivity assay on the Qubit 3·0 instrument (Life Technologies, USA). Amplicons from each sample were normalized, pooled, and barcoded using the Rapid Barcoding Kit 96 kit (EXP-NBD 196, Oxford Nanopore Technologies, UK). Next, sequencing libraries were generated using the SQK-LSK109 Kit (Oxford Nanopore Technologies, Oxford, UK) and were loaded onto an R9·4·1 flow-cell (Oxford Nanopore Technologies, UK). Then, FAST5 files containing the raw signal data were base-called, demultiplexed, and trimmed using Guppy version 4·4·1 (Oxford Nanopore Technologies, UK). The reads were aligned against the CHIKV strain C302F/2016/BR (GenBank accession no. KY055011) using minimap2 version 2·17·r941 (2) and converted to a sorted BAM file using SAMtools (3). Length filtering, quality testing, and primmer trimming were performed for each barcode using guppyplex. Variants were detected with medaka_variants and the consensus sequence were built using margin_medaka_consensus (Oxford Nanopore Technologies, UK). Genome regions with a depth coverage below 20-fold were represented with “N” characters.

Supplementary Figures

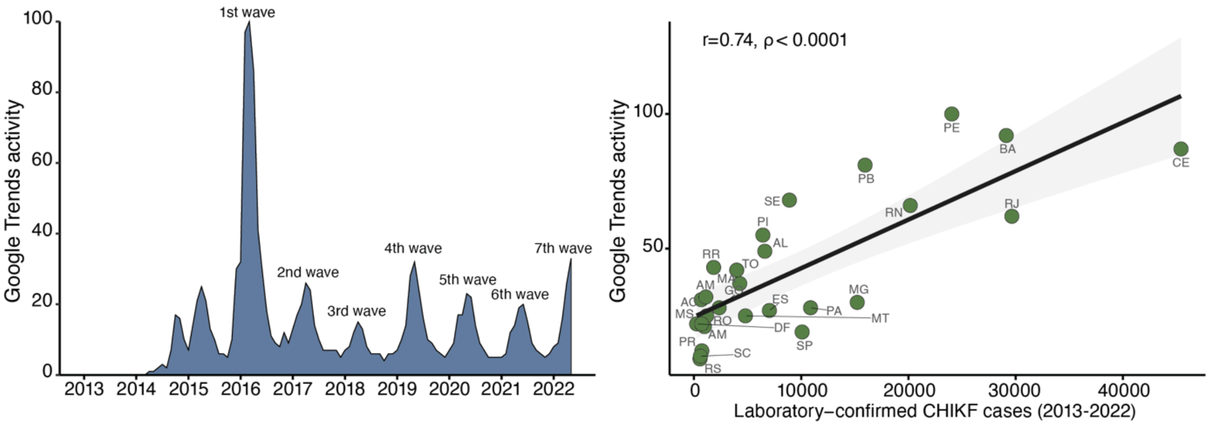

Supplementary Figure 1. Digital surveillance of chikungunya in Brazil. Timeline of Google Trends activity for the term "chikungunya" in Brazil from January 2013 to May 2022 (left). Correlation between Google Trends activity and laboratory confirmed chikungunya cases per state from January 2013 to May 2022 (right). The correlation was calculated using the Spearman's rank correlation coefficient. AC, Acre; AL, Alagoas; AM, Amazonas; AP, Amapá; BA, Bahia; CE, Ceará; DF, Distrito Federal; ES, Espírito Santo; GO, Goiás; MA, Maranhão; MG, Minas Gerais; MS, Mato Grosso do Sul; MT, Mato Grosso; PA, Pará; PB, Paraíba; PE, Pernambuco; PI, Piauí; PR, Paraná; RJ, Rio de Janeiro; RN, Rio Grande do Norte; RO, Rondônia; RR, Roraima; RS, Rio Grande do Sul; SC, Santa Catarina; SE, Sergipe; SP, São Paulo; TO, Tocantins.

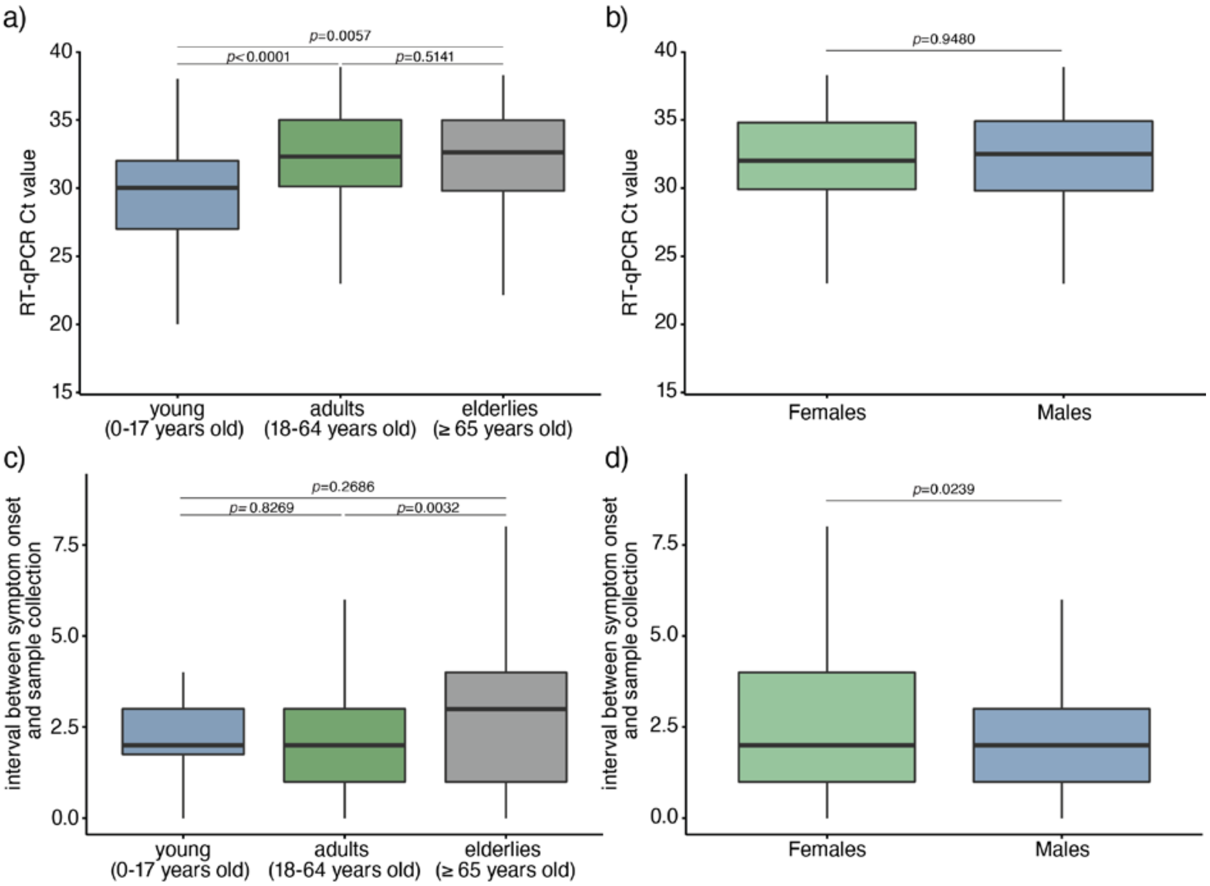

Supplementary Figure 2. Distribution of cycle threshold values and the interval between symptom onset and sample collection at a Laboratory of Public Health of Ceará State. Cycle threshold values for chikungunya cases were stratified according to the age group (**a**) or sex (**b**). The interval between symptom onset and sample collection per age group (**c**) or sex (**d**). The statistical difference between sexes in all age groups was calculated by One-way ANOVA with the Tukey's HSD (honestly significant difference) test. The significance level was determined as a p-value less than 0.05.

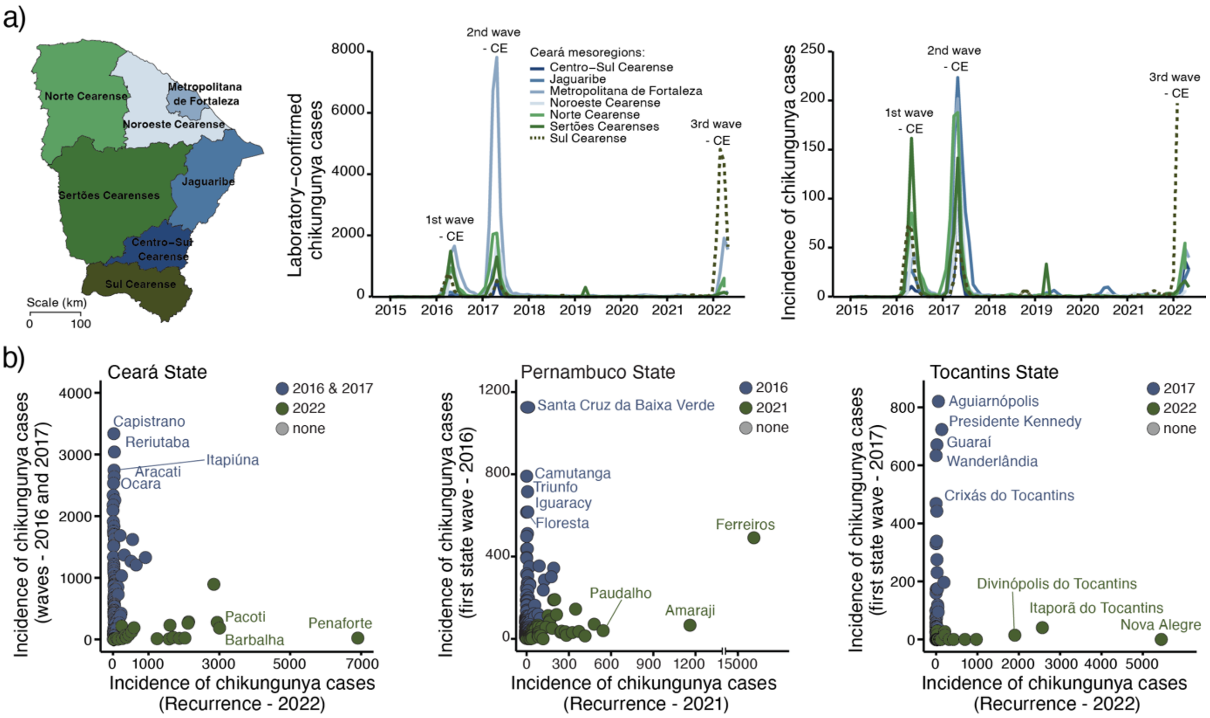

Supplementary Figure 3. Spatiotemporal distribution of chikungunya recurrence in Brazil. **a)** Map of mesoregions of Ceará State, Brazil (left). Number of chikungunya cases (center) and chikungunya incidence (right) per mesoregions of Ceará State from 1 January 2015 to 31 May 2022. **b)** Comparison of chikungunya incidence caused by the previous chikungunya waves and recurrence in Ceará (left), Tocantins (center), and Pernambuco States (right).

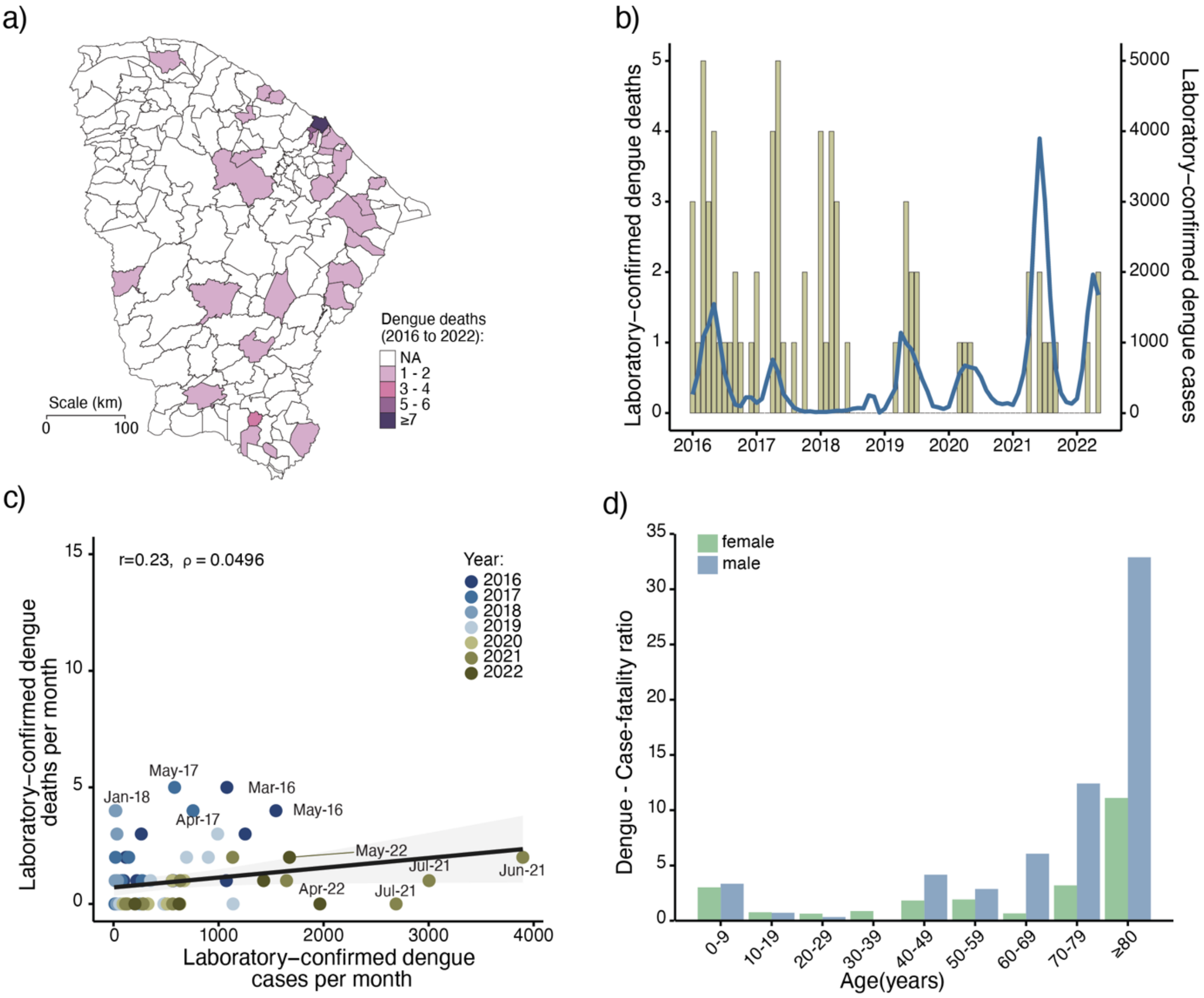

Supplementary Figure 4. Dengue deaths in Ceará State, Brazil. a) Spatial distribution of laboratory-confirmed dengue fever deaths per municipality in Ceará State from 2016 to 2022. **b)** Number of laboratory-confirmed DENV deaths (blue line) and cases (gold bars) per month from 1 January 2016 to 31 May 2022. **b)** The Pearson's correlation coefficients of laboratory-confirmed dengue deaths and laboratory-confirmed dengue cases per month from 2016 to 2022 in Ceará State. **d)** The accumulate case fatality ratio by age-sex groups from dengue from 2016 to 2022 in Ceará State.

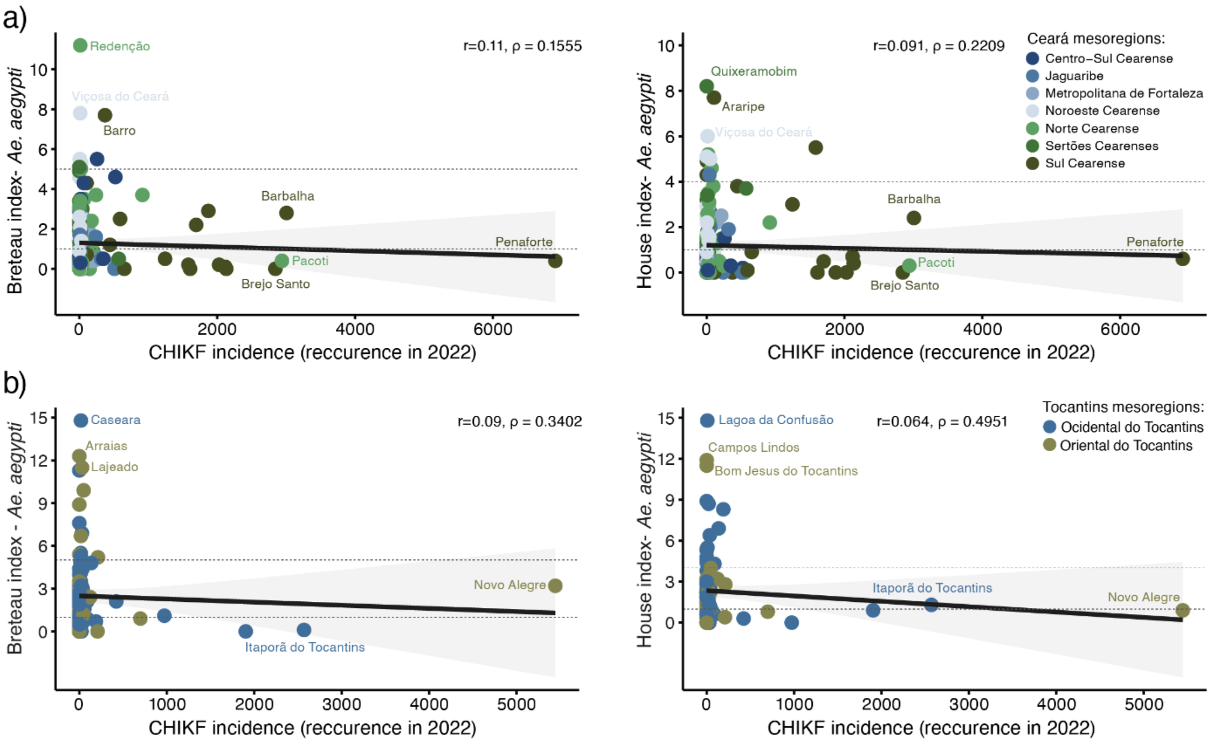

Supplementary Figure 5. Correlation between *Ae. aegypti* population density indices and chikungunya incidence in Ceará and Tocantins States in 2022. Correlation coefficients of Breteau index (left) and House index (right) per chikungunya incidence in 2022 in Ceará State municipalities (**a**) and Tocantins State municipalities (**b**). The correlation was calculated using Spearman's rank correlation coefficient. The circles were colored based on mesoregions. The upper dash line indicates the risk situation (Breteau index = 5 and House index = 4), and the bottom dash line denotes the alert situation (Breteau index and House index > 1) according to the National Dengue Control Programme in Brazil (4).

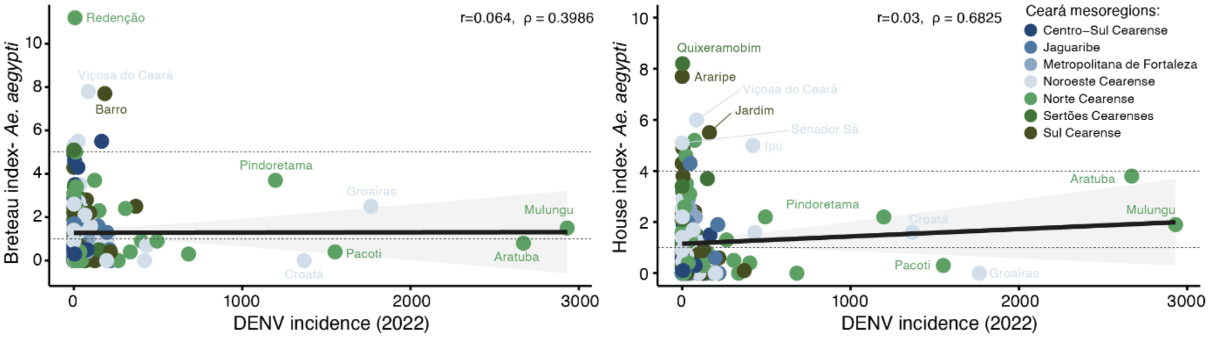

Supplementary Figure 6. Correlation between *Ae. aegypti* population density indices and dengue incidence in Ceará in 2022. Correlation coefficients of Breteau index (left) and House index (right) per dengue incidence in 2022. The correlation was calculated using Spearman's rank correlation coefficient. The circles were colored based on Ceará mesoregions. The upper dash line indicates the risk situation (Breteau index = 5 and House index = 4), and the bottom dash line denotes the alert situation (Breteau index and House index > 1) according to the National Dengue Control Programme in Brazil (4).

Table Supplementary 1. Information of chikungunya cases sequenced in this study.

ID	Sample	Age (years)	Sex	Municipality	Onset symptoms	Sample collection	Ct value - RT-PCR	GenBank
1	serum	45	M	Barbalha	09-Feb-22	10-Feb-22	23.3	OP964932
3	serum	11	F	Barbalha	08-Feb-22	10-Feb-22	21.94	OP964933
4	serum	29	F	Barbalha	09-Feb-22	10-Feb-22	29.55	OP964934
5	serum	27	M	Barbalha	09-Feb-22	10-Feb-22	25.14	OP964935
6	serum	59	F	Barbalha	06-Feb-22	10-Feb-22	29.03	OP964936
7	serum	59	M	Barbalha	09-Feb-22	10-Feb-22	23.14	OP964937
8	serum	24	M	Barbalha	09-Feb-22	10-Feb-22	25.36	OP964938
9	serum	57	M	Barbalha	09-Feb-22	10-Feb-22	26.15	OP964939
10	serum	20	M	Barbalha	10-Feb-22	10-Feb-22	25.49	OP964940
11	serum	55	F	Barbalha	10-Feb-22	10-Feb-22	21.63	OP964941
12	serum	56	F	Barbalha	10-Feb-22	10-Feb-22	23.33	OP964942
13	serum	27	M	Barbalha	11-Feb-22	15-Feb-22	22.9	OP964943
14	serum	31	M	Barbalha	14-Feb-22	15-Feb-22	28.96	OP964944
15	serum	46	F	Barbalha	12-Feb-22	15-Feb-22	27.69	OP964945
16	serum	74	M	Barbalha	13-Feb-22	15-Feb-22	22.13	OP964946
17	serum	46	F	Barbalha	19-Feb-22	21-Feb-22	29.35	OP964947
18	serum	12	M	Barbalha	19-Feb-22	21-Feb-22	27.17	OP964948
20	serum	27	F	Barbalha	14-Feb-22	17-Feb-22	28.49	OP964949
21	serum	64	F	Barbalha	15-Feb-22	18-Feb-22	27.73	OP964950
22	serum	43	F	Barbalha	16-Feb-22	18-Feb-22	29.68	OP964951
23	serum	68	M	Barbalha	14-Feb-22	18-Feb-22	26.49	OP964952
24	serum	11	F	Barbalha	14-Feb-22	18-Feb-22	29.27	OP964953
25	serum	46	F	Fortaleza	02-Mar-22	04-Mar-22	29.6	OP964954
45	serum	63	M	Fortaleza	06-Mar-22	07-Mar-22	28.8	OP964955
46	serum	36	F	Pacoti	28-Feb-22	04-Mar-22	30.82	OP964956
47	serum	41	F	Pacoti	07-Mar-22	08-Mar-22	30.64	OP964957
48	serum	31	M	Pacoti	07-Mar-22	08-Mar-22	30.63	OP964958
50	serum	6	F	Pacoti	06-Mar-22	09-Mar-22	29.13	OP964959
52	cerebrospinal fluid	10 days	M	Fortaleza	25-Feb-22	03-Mar-22	29.6	OP964960
53	serum	1 month	F	Barbalha	17-Feb-22	22-Feb-22	24.51	OP964961
54	serum	77	F	Pacoti	25-Feb-22	28-Feb-22	28.68	OP964962
55	serum	49	M	Barbalha	22-Feb-22	23-Feb-22	29.94	OP964963
56	serum	68	F	Barbalha	21-Feb-22	22-Feb-22	29.93	OP964964
57	serum	37	F	Pacoti	06-Feb-22	07-Feb-22	29.59	OP964964
58	serum	62	M	Barbalha	11-Feb-22	15-Feb-22	18	OP964966
59	serum	10	F	Barbalha	14-Feb-22	16-Feb-22	23	OP964967
60	serum	53	F	Barbalha	14-Feb-22	15-Feb-22	20	OP964968
61	serum	83	M	Barbalha	13-Feb-22	15-Feb-22	21	OP964969
62	serum	43	M	Barbalha	14-Feb-22	15-Feb-22	24	OP964970

63	serum	46	F	Barbalha	13-Feb-22	14-Feb-22	27	OP964971
64	serum	5	M	Barbalha	14-Feb-22	16-Feb-22	20	OP964972
65	serum	33	F	Barbalha	13-Feb-22	16-Feb-22	20	OP964973
66	serum	76	M	Barbalha	12-Feb-22	16-Feb-22	17	OP964974
67	serum	46	F	Barbalha	14-Feb-22	17-Feb-22	21	OP964975
68	serum	37	F	Penaforte	22-Feb-22	24-Feb-22	20	OP964976
70	serum	66	F	Juazeiro do Norte	03-Mar-22	07-Mar-22	28	OP964977
72	serum	72	F	Juazeiro do Norte	03-Mar-22	07-Mar-22	18	OP964978
73	serum	39	F	Farias Brito	03-Mar-22	07-Mar-22	25	OP964979
74	serum	8	F	Juazeiro do Norte	03-Mar-22	08-Mar-22	23	OP964980
75	serum	73	F	Juazeiro do Norte	03-Mar-22	08-Mar-22	19	OP964981
76	serum	22	F	Juazeiro do Norte	03-Mar-22	08-Mar-22	27	OP964982
77	serum	40	F	Juazeiro do Norte	03-Mar-22	08-Mar-22	21	OP964983
78	serum	36	F	Juazeiro do Norte	03-Mar-22	08-Mar-22	23	OP964984
79	serum	74	F	Juazeiro do Norte	03-Mar-22	08-Mar-22	21	OP964985
80	serum	3	M	Juazeiro do Norte	07-Mar-22	09-Mar-22	20	OP964986
81	serum	65	M	Juazeiro do Norte	03-Mar-22	09-Mar-22	30	OP964987
82	serum	60	M	Juazeiro do Norte	03-Mar-22	09-Mar-22	25	OP964988
83	serum	77	M	Juazeiro do Norte	03-Mar-22	09-Mar-22	20	OP964989
84	serum	57	F	Juazeiro do Norte	03-Mar-22	09-Mar-22	22	OP964990
85	serum	37	F	Juazeiro do Norte	03-Mar-22	09-Mar-22	21	OP964991
86	serum	23	M	Juazeiro do Norte	03-Mar-22	09-Mar-22	24	OP964992

Legend: F, female. M, male.

Table Supplementary 2. Chikungunya epidemic waves in Brazil.

Wave	Year	Total cases[1]	States most affected	Number case in wave	Period of CHIFV epidemic peak	Peak of CHIFV cases[3]
1	2016	44,604	Pernambuco	8,794 (19.7%)[2]	February to March	539 to 758
			Ceará	11,474 (27.7%)[2]	April to June	575 to 835
2	2017	41,974	Ceará	21,486 (51.2%)[2]	March to July	535 to 1,692
3	2018	24,097	Rio de Janeiro	6,316 (26.2%)[2]	April	329 to 392
4	2019	33,740	Rio de Janeiro	15,984 (47.4%)[2]	April to June	581 to 1,087
5	2020	31,233	Bahia	12,390 (39.7%)[2]	May to June	562 to 620
6	2021	39,224	Pernambuco	6,628 (16.9%)[2]	June to July	311 to 407
			São Paulo	6,539 (16.7%)[2]	March to April	304 to 390
7	2022	35,183	Ceará	8,413 (23.9%)[2]	March to May	≥ 500*

Legend: [1]Total of chikungunya cases reported in Brazil to the Ministry of Health. [2]Percentage of chikungunya cases in the state related to the total cases in the wave. [3] Number of chikungunya cases per epidemiological week in the peak of epidemic in the state. *Data available up to May 2022.

Table Supplementary 3. Probability of chikungunya across sexes and age-groups in Ceará State from 2016 to 2022.

Groups	Odds ratio (95% CI)	p-value
Male	0·87 (0·85 - 0·89)	<0·0001
18-39 years old	1·32 (1·27 - 1·36)	<0·0001
40-54 years old	1·71 (1·65 - 1·77)	<0·0001
55-74 years old	2·05 (1·98 - 2·13)	<0·0001
≥75 years old	2·33 (2·18 - 2·49)	<0·0001

Logistic regression using the dataset of all individuals tested for chikungunya in Ceará State from 2016 to 2022. Reference group for sex is female, and the age-group <18 years old was used as reference age-group. The significance level was determined as a p-value less than 0.05. 95% CI, 95% confidence interval (lower and upper).

Table Supplementary 4. Probability of chikungunya-related death across sexes and age-groups in Ceará State from 2016 to 2022.

Groups	Odds ratio (95% CI)	p-value
Male	0·73 (0·46 - 1·15)	0·1776
18-39 years old	1·71 (0·81 - 3·6)	0·1571
40-54 years old	1·71 (0·82 - 3·53)	0·1503
55-74 years old	2·28 (1·11 - 4·68)	0·0249
≥75 years old	4·01 (1·83 - 8·78)	0·0005

Logistic regression using the dataset of all deaths tested for chikungunya in Ceará State from 2016 to 2022. Reference group for sex is female and the age-group <18 years old was used as reference age-group. The significance level was determined as a p-value less than 0.05. 95% CI, 95% confidence interval (lower and upper).

Table 5. Dataset of Brazilian chikungunya virus complete coding sequences classified into East-Central-South-African genotype and available in GenBank.

GenBank	Country	State	Date
MG649984	Brazil	Rio de Janeiro	2015
MG649985	Brazil	Rio de Janeiro	2015
KP164568	Brazil	Bahia	26-Aug-14
KP164569	Brazil	Bahia	28-Aug-14
KP164570	Brazil	Bahia	3-Sep-14
MK121891	Brazil	Amazonas	15-Jul-15
MK121892	Brazil	Amazonas	15-Jul-15
MK121893	Brazil	Amazonas	15-Jul-15
KU940226	Brazil	Bahia	1-Aug-15
MK993756	Brazil	Rio Grande do Norte	14-Jan-16
MK121894	Brazil	Amazonas	30-Jan-16
MH000700	Brazil	Pernambuco	5-Feb-16
MH000702	Brazil	Pernambuco	15-Feb-16

MH000703	Brazil	Pernambuco	15-Feb-16
MH000706	Brazil	Pernambuco	18-Feb-16
MH000704	Brazil	Pernambuco	19-Feb-16
MK244635	Brazil	Rio de Janeiro	19-Feb-16
KY055011	Brazil	Sergipe	20-Feb-16
MH000705	Brazil	Pernambuco	25-Feb-16
KY124328	Brazil	Rio de Janeiro	16-Mar-16
KY124329	Brazil	Rio de Janeiro	16-Mar-16
MG649983	Brazil	Rio de Janeiro	28-Mar-16
MG649970	Brazil	Rio de Janeiro	29-Mar-16
MG649976	Brazil	Rio de Janeiro	29-Mar-16
KY704933	Brazil	Alagoas	30-Mar-16
KY704935	Brazil	Alagoas	1-Apr-16
KY704936	Brazil	Alagoas	1-Apr-16
MN783352	Brazil	Bahia	1-Apr-16
MK244632	Brazil	Rio de Janeiro	5-Apr-16
MK244638	Brazil	Rio de Janeiro	5-Apr-16
OM128439	Brazil	Rio de Janeiro	6-Apr-16
KY704949	Brazil	Alagoas	7-Apr-16
KY704950	Brazil	Alagoas	7-Apr-16
KY704952	Brazil	Alagoas	7-Apr-16
MG649974	Brazil	Rio de Janeiro	8-Apr-16
KY704953	Brazil	Alagoas	9-Apr-16
KY704940	Brazil	Alagoas	14-Apr-16
KY704941	Brazil	Alagoas	14-Apr-16
KY704945	Brazil	Alagoas	14-Apr-16
KY704947	Brazil	Alagoas	15-Apr-16
KY704942	Brazil	Alagoas	16-Apr-16
KY704939	Brazil	Alagoas	17-Apr-16
KY704938	Brazil	Alagoas	19-Apr-16
KY704948	Brazil	Alagoas	19-Apr-16
MK244639	Brazil	Rio de Janeiro	19-Apr-16
MG649980	Brazil	Rio de Janeiro	27-Apr-16
MK244634	Brazil	Rio de Janeiro	27-Apr-16
OM128438	Brazil	Rio de Janeiro	29-Apr-16
MK244636	Brazil	Rio de Janeiro	2-May-16
MG649972	Brazil	Rio de Janeiro	5-May-16
MK244633	Brazil	Rio de Janeiro	6-May-16
MK244637	Brazil	Rio de Janeiro	10-May-16
KY704955	Brazil	Paraíba	17-Jun-16
KY704954	Brazil	Paraíba	20-Jun-16
KU940225	Brazil	Bahia	15-Jul-16

MG649975	Brazil	Rio de Janeiro	27-Jul-16
MG649977	Brazil	Rio de Janeiro	28-Jul-16
MG649981	Brazil	Rio de Janeiro	28-Jul-16
MG649971	Brazil	Rio de Janeiro	22-Aug-16
MN783353	Brazil	Bahia	13-Oct-16
MG649973	Brazil	Rio de Janeiro	12-Dec-16
MT526900	Brazil	Pará	24-Jan-17
MT526900	Brazil	Pará	25-Jan-17
MT526901	Brazil	Pará	25-Jan-17
MT526902	Brazil	Pará	25-Jan-17
MT526903	Brazil	Pará	26-Jan-17
MT526904	Brazil	Pará	26-Jan-17
MK121897	Brazil	Roraima	20-Feb-17
MK121898	Brazil	Roraima	22-Feb-17
MK121904	Brazil	Roraima	2-Mar-17
MK121905	Brazil	Roraima	2-Mar-17
MK121896	Brazil	Roraima	3-Mar-17
MK121908	Brazil	Roraima	5-Mar-17
MK244640	Brazil	Rio de Janeiro	7-Mar-17
MK244641	Brazil	Rio de Janeiro	9-Mar-17
MK121903	Brazil	Roraima	15-Mar-17
MG649978	Brazil	Rio de Janeiro	16-Mar-17
MK121899	Brazil	Roraima	17-Mar-17
MK121900	Brazil	Roraima	17-Mar-17
MK121901	Brazil	Roraima	17-Mar-17
MK121902	Brazil	Roraima	17-Mar-17
MT877207	Brazil	Ceará	19-Mar-17
MK121895	Brazil	Amazonas	20-Mar-17
MT877208	Brazil	Ceará	22-Mar-17
MG649982	Brazil	Rio de Janeiro	24-Mar-17
MK121906	Brazil	Roraima	27-Mar-17
MK121907	Brazil	Roraima	27-Mar-17
MT877210	Brazil	Ceará	14-Apr-17
MK518395	Brazil	Maranhão	16-May-17
MT877211	Brazil	Ceará	3-Jun-17
MK244642	Brazil	Rio de Janeiro	18-Feb-18
OM128440	Brazil	Rio de Janeiro	20-Mar-18
MK244644	Brazil	Rio de Janeiro	26-Mar-18
OM128432	Brazil	Rio de Janeiro	26-Mar-18
MK244647	Brazil	Rio de Janeiro	27-Mar-18
MK244643	Brazil	Rio de Janeiro	28-Mar-18
MK244645	Brazil	Rio de Janeiro	28-Mar-18

MK244646	Brazil	Rio de Janeiro	28-Mar-18
MK244649	Brazil	Rio de Janeiro	3-Apr-18
MK244650	Brazil	Rio de Janeiro	3-Apr-18
MK244648	Brazil	Rio de Janeiro	4-Apr-18
MK244651	Brazil	Rio de Janeiro	5-Apr-18
MK244652	Brazil	Rio de Janeiro	5-Apr-18
MK244653	Brazil	Rio de Janeiro	6-Apr-18
MK244655	Brazil	Rio de Janeiro	6-Apr-18
MK244656	Brazil	Rio de Janeiro	6-Apr-18
OM128436	Brazil	Rio de Janeiro	5-Jun-18
OM128431	Brazil	Rio de Janeiro	6-Jun-18
OM128433	Brazil	Rio de Janeiro	6-Jun-18
OM128434	Brazil	Rio de Janeiro	7-Jun-18
OM128437	Brazil	Rio de Janeiro	7-Jun-18
MK156054	Brazil	Bahia	21-Sep-18
MK156055	Brazil	Bahia	21-Sep-18
MK156056	Brazil	Bahia	21-Sep-18
MK156058	Brazil	Bahia	21-Sep-18
MK156059	Brazil	Bahia	21-Sep-18
MK156060	Brazil	Bahia	26-Sep-18
MK156061	Brazil	Bahia	26-Sep-18
MK156062	Brazil	Bahia	26-Sep-18
MK156063	Brazil	Bahia	26-Sep-18
MK156064	Brazil	Bahia	26-Sep-18
MW260512	Brazil	Rio Grande do Norte	23-Mar-19
MW260513	Brazil	Rio Grande do Norte	23-Mar-19
MW260515	Brazil	Rio Grande do Norte	28-Mar-19
MW260516	Brazil	Rio Grande do Norte	28-Mar-19
MW260518	Brazil	Rio Grande do Norte	30-Mar-19
OL898663	Brazil	São Paulo	5-Sep-20
OL898714	Brazil	São Paulo	20-Nov-20
OL898669	Brazil	São Paulo	17-Dec-20
OL898675	Brazil	São Paulo	26-Dec-20
OL898668	Brazil	São Paulo	6-Jan-21
OL898682	Brazil	São Paulo	7-Jan-21
OL898707	Brazil	São Paulo	7-Jan-21
OL898689	Brazil	São Paulo	15-Jan-21
OL898690	Brazil	São Paulo	16-Jan-21
OL898674	Brazil	São Paulo	23-Jan-21
OL898696	Brazil	São Paulo	28-Jan-21
OL898700	Brazil	São Paulo	28-Jan-21
OL898704	Brazil	São Paulo	28-Jan-21

OL898664	Brazil	São Paulo	31-Jan-21
OL898683	Brazil	São Paulo	31-Jan-21
OL898667	Brazil	São Paulo	1-Feb-21
OL898670	Brazil	São Paulo	2-Feb-21
OL898676	Brazil	São Paulo	2-Feb-21
OL898710	Brazil	São Paulo	2-Feb-21
OL898711	Brazil	São Paulo	8-Feb-21
OL898697	Brazil	São Paulo	9-Feb-21
OL898699	Brazil	São Paulo	9-Feb-21
OL898706	Brazil	São Paulo	9-Feb-21
OL898677	Brazil	São Paulo	13-Feb-21
OL898693	Brazil	São Paulo	13-Feb-21
OL898705	Brazil	São Paulo	13-Feb-21
OL898684	Brazil	São Paulo	14-Feb-21
OL898671	Brazil	São Paulo	16-Feb-21
OL898712	Brazil	São Paulo	18-Feb-21
OL898681	Brazil	São Paulo	20-Feb-21
OL898691	Brazil	São Paulo	21-Feb-21
OL898665	Brazil	São Paulo	22-Feb-21
OL898685	Brazil	São Paulo	22-Feb-21
OL898666	Brazil	São Paulo	25-Feb-21
OL898672	Brazil	São Paulo	25-Feb-21
OL898698	Brazil	São Paulo	25-Feb-21
OL898678	Brazil	São Paulo	5-Mar-21
OL898692	Brazil	São Paulo	9-Mar-21
OL898673	Brazil	São Paulo	15-Mar-21
OL898680	Brazil	São Paulo	22-Mar-21
OL898688	Brazil	São Paulo	23-Mar-21
OL898686	Brazil	São Paulo	25-Mar-21
OL898703	Brazil	São Paulo	6-Apr-21
OL898695	Brazil	São Paulo	11-Apr-21
OL898679	Brazil	São Paulo	13-Apr-21
OL898687	Brazil	São Paulo	16-Apr-21
OL898702	Brazil	São Paulo	27-Apr-21
OL898713	Brazil	São Paulo	29-Apr-21
OL898701	Brazil	São Paulo	5-May-21
OL898708	Brazil	São Paulo	5-May-21
OL898715	Brazil	São Paulo	6-May-21
OL898694	Brazil	São Paulo	11-May-21
OL898709	Brazil	São Paulo	12-May-21
MG649979	Brazil	Rio de Janeiro	Mar-17

References

1. Quick J, Grubaugh ND, Pullan ST, Claro IM, Smith AD, Gangavarapu K, et al. Multiplex PCR method for MinION and Illumina sequencing of Zika and other virus genomes directly from clinical samples. Nat Protoc. 2017;12(6):1261-76.
2. Li H. Minimap2: pairwise alignment for nucleotide sequences. Bioinformatics. 2018;34(18):3094-100.
3. Li H, Handsaker B, Wysoker A, Fennell T, Ruan J, Homer N, et al. The Sequence Alignment/Map format and SAMtools. Bioinformatics. 2009;25(16):2078-9.
4. Ministério da Saúde do Brasil. Programa Nacional de Controle da Dengue. Brasília, 2002.
